# Utilizing machine learning algorithms for the prediction of carotid artery plaques in a Chinese population

**DOI:** 10.3389/fphys.2023.1295371

**Published:** 2023-11-06

**Authors:** Shuwei Weng, Jin Chen, Chen Ding, Die Hu, Wenwu Liu, Yanyi Yang, Daoquan Peng

**Affiliations:** ^1^ Department of Cardiovascular Medicine, The Second Xiangya Hospital, Central South University, Changsha, Hunan, China; ^2^ Research Institute of Blood Lipid and Atherosclerosis, Changsha, Hunan, China; ^3^ Department of Cardiology, Suzhou Dushu Lake Hospital, Dushu Lake Hospital Affiliated to Soochow University, Medical Center of Soochow University, Suzhou, Jiangsu, China; ^4^ Health Management Center, The Second Xiangya Hospital, Central South University, Changsha, Hunan, China

**Keywords:** carotid artery plaque, machine learning, LightGBM, screening, primary prevention

## Abstract

**Background:** Ischemic stroke is a significant global health issue, imposing substantial social and economic burdens. Carotid artery plaques (CAP) serve as an important risk factor for stroke, and early screening can effectively reduce stroke incidence. However, China lacks nationwide data on carotid artery plaques. Machine learning (ML) can offer an economically efficient screening method. This study aimed to develop ML models using routine health examinations and blood markers to predict the occurrence of carotid artery plaques.

**Methods:** This study included data from 5,211 participants aged 18–70, encompassing health check-ups and biochemical indicators. Among them, 1,164 participants were diagnosed with carotid artery plaques through carotid ultrasound. We constructed six ML models by employing feature selection with elastic net regression, selecting 13 indicators. Model performance was evaluated using accuracy, sensitivity, specificity, Positive Predictive Value (PPV), Negative Predictive Value (NPV), F1 score, kappa value, and Area Under the Curve (AUC) value. Feature importance was assessed by calculating the root mean square error (RMSE) loss after permutations for each variable in every model.

**Results:** Among all six ML models, LightGBM achieved the highest accuracy at 91.8%. Feature importance analysis revealed that age, Low-Density Lipoprotein Cholesterol (LDL-c), and systolic blood pressure were important predictive factors in the models.

**Conclusion:** LightGBM can effectively predict the occurrence of carotid artery plaques using demographic information, physical examination data and biochemistry data.

## 1 Introduction

2019 Global Burden of Disease Study reported that there were approximately 101 million cases of stroke worldwide, with approximately 6.65 million deaths attributed to stroke. Stroke ranked as the third leading cause of death and disability-adjusted life years globally, following neonatal diseases and ischemic heart disease. It was also the second leading global cause of death in 2019 (GBD, 2019 Stroke Collaborators, 2021). Ischemic stroke, as one of the major subtypes of stroke, stands out as a crucial neurovascular cause of mortality and disability. Research suggests that by 2023, the global number of deaths due to ischemic stroke may increase from 3.29 million in 2019 to approximately 4.9 million (Fan et al., 2023). In 2016, China incurred an estimated $12.2 billion in hospitalization costs for stroke, imposing a significant burden on both the economy and healthcare resources (Qi et al., 2020).

Carotid artery plaque, as an independent risk indicator for stroke beyond classical risk factors (Prati et al., 2008), increases the risk of stroke by 13%–18% for every 0.1 mm increase in intima-media thickness of the carotid artery (Lorenz et al., 2007). Up to 25% of ischemic cerebrovascular events are attributed to carotid atherosclerosis (Saba et al., 2022). Early screening and prevention of carotid artery plaques can reduce the risk of stroke, thereby mitigating its adverse impacts on individuals, families, and society. However, China lacks a nationwide epidemiological survey on the prevalence of carotid artery plaques. This gap may be due to carotid ultrasound being a preventive health risk assessment rather than the definitive diagnostic criterion for a severe illness. Hence, there’s an urgent need to develop a cost-effective screening method for carotid artery plaques.

With the significant improvement in computer performance, machine learning possesses the ability to handle large-scale and high-dimensional data, which traditional statistics may not handle effectively. Machine learning can automatically extract complex patterns and associations from data without the need for explicit *a priori* assumptions. Through iterative optimization on vast data, it enhances model generalization and applies to complex real-world situations. Machine learning has already found applications in medical diagnoses for various diseases, such as fatty liver and diabetes (Joshi and Dhakal, 2021; Weng et al., 2023).

In previous machine learning studies related to carotid artery plaques, most utilized convolutional neural networks based on segmentation and texture feature extraction from carotid ultrasound images to predict plaque properties or distinguish plaque components. For example, Latha et al. (2021) achieved prediction models for carotid artery plaques with accuracies of 84.21%, 88.64%, and 91.41% using decision trees, logistic regression, and random forests, respectively, based on 22 features extracted from carotid ultrasound images. Lekadir et al. (2017) constructed a convolutional neural network model using approximately 90,000 plaque images and corresponding features. This model accurately predicted plaque components such as lipid core, fibrous cap, and calcification areas, achieving a clinical relevance of 0.90.

Although the models constructed based on ultrasound images have demonstrated high predictive efficiency and plaque component recognition ability, the manpower and material costs for widespread screening of carotid artery plaques are high. In contrast, utilizing physical examinations and routine biochemical indicators as features for machine learning models offers advantages such as convenient data collection without additional examination costs. Therefore, the main purpose of this study is to build suitable and highly accurate machine learning models based on these indicators to predict plaque occurrence.

## 2 Materials and methods

### 2.1 Study data

**FIGURE 1:**
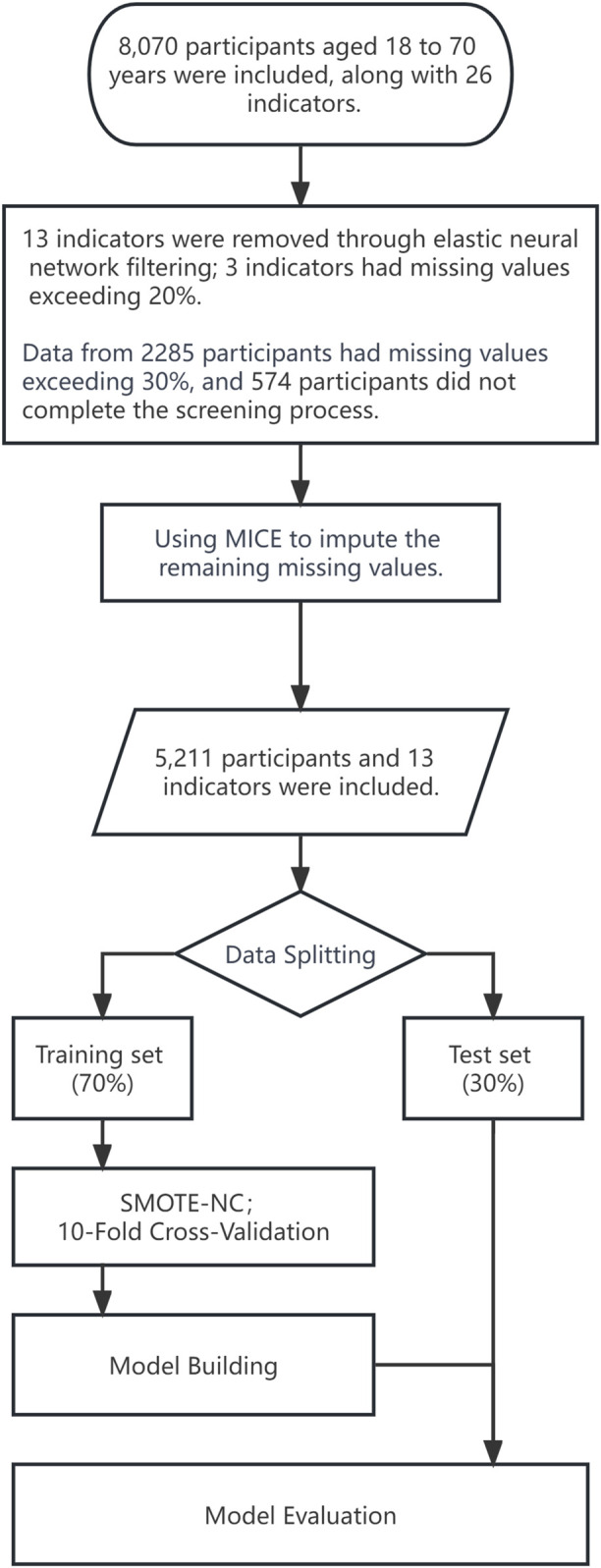
Data processing flowchart. MICE, multiple imputation by chained equations; SMOTE-NC, synthetic minority over-sampling technique for nominal continuous.

The dataset used in this study was provided by the Health Management Center of Xiangya Second Hospital, Central South University, Changsha, China, and encompassed data from 8,070 patients (Figure 1). Participants aged between 18 and 70 were recruited from June 2018 to December 2020. The data collection process meticulously avoided including any sensitive information. The dataset was limited to 29 variables, including health examination data, age, and blood biochemical indicators. To ensure data integrity during the construction of machine learning models, samples with missing data exceeding 30% (2,285 cases) or incomplete carotid artery color ultrasound screening (574 cases) were excluded from the dataset. For the remaining samples with partial missing values, imputation was performed using multiple imputation by chained equations (Supplementary Figure S1). In this study, the diagnosis of carotid artery plaques was based on the results of carotid artery ultrasound imaging. According to the Chinese Health Checkup Guidelines for Carotid Artery Ultrasonography (Chinese Society of Health Management and Chinese Society of Ultrasound in Medicine CSoC, 2015), released in 2015, patients are considered positive for carotid artery plaques if the perpendicular distance between the leading edge of the intima-lumen interface to the leading edge of the media-adventitia interface is greater than 1.5 mm. This distance should be larger by at least 0.5 mm compared to the surrounding normal values, or it should exceed the surrounding normal values by more than 50%. Additionally, patients showing localized structural changes that protrude into the lumen are also categorized as positive for carotid artery plaques. The ultrasound machine utilized in this study was the GE LOGIQ E9. All diagnostic outcomes from carotid artery ultrasound examinations were conducted by attending physicians at our hospital’s imaging center, and they underwent validation by experienced medical professionals.

### 2.2 Data processing

Feature selection stands as a pivotal process within classification prediction models, given its profound impact on model performance. The primary aim of feature selection is to identify the most relevant subset of features that can significantly enhance classification accuracy. Among the 13 feature variables listed in Table 1, the selection of these variables is informed by several factors. These encompass commonly used indicators with clinical significance for predicting carotid artery plaques, the incorporation of supplementary variables to augment feature diversity, and the utilization of a variable selection approach grounded in elastic net regression. Elastic Net regression is a widely utilized linear regression technique in feature engineering. It exhibits insensitivity to feature scaling, enabling its adaptability to features of varying scales without necessitating additional feature scaling operations. This method amalgamates the attributes of Lasso regression and Ridge regression. By incorporating both L1 and L2 regularization terms into its loss function, it strikes a balance between feature selection and parameter adjustment. The L1 penalty fosters sparsity by effectively shrinking certain coefficients to zero, thereby facilitating feature selection. Meanwhile, the L2 penalty regulates model complexity to prevent overfitting. In scenarios featuring multiple highly correlated features, Elastic Net regression aptly shrinks their weights, mitigating the influence of multicollinearity. This contributes to enhancing model stability and generalizability. In this study, an Elastic Net regression model was constructed. The process involved a thorough exploration of the alpha parameter ranging from 0 to 1, with increments of 0.05. The selection of the optimal alpha value, indicative of the best performance, was achieved by utilizing cross-validation with mean squared error as the performance metric. Subsequently, based on the determined alpha value, the coefficients of the feature variables within the Elastic Net regression model were employed for feature selection.

**TABLE 1:** Characteristics of study population.

Characteristic	Absence of carotid plaques (*N* = 4,047)		Presence of carotid plaques (*N* = 1,164)		*p*-value
	(Mean/number)	(SD/proportion)	(Mean/number)	(SD/proportion)	
Age	48.11	9.11	57.88	7.07	<0.001
Sex					<0.001
Female	1221	30.20%	266	22.90%	
Male	2826	69.80%	898	77.10%	
Sp	121.23	15.77	129.49	16.58	<0.001
BMI	24.73	3.19	25.00	3.06	0.008
Fbg	5.10	1.13	5.48	1.58	<0.001
TC	4.90	0.92	5.02	1.00	<0.001
HDL-c	1.28	0.31	1.27	0.31	0.45
LDL-c	2.97	0.82	3.17	0.89	<0.001
Lp(a)	199.59	217.28	221.94	258.91	0.007
ALT	26.71	17.24	24.59	14.71	<0.001
BUN	5.13	1.24	5.56	1.36	<0.001
Cr	72.29	15.09	75.38	16.15	<0.001
UA	343.07	84.36	352.19	85.85	0.001

Sp, systolic pressure; BMI, body mass index; Fbg, fasting blood-glucose; TC, total cholesterol; HDL-c, high-density lipoprotein cholesterol; LDL-c, low-density lipoprotein cholesterol; Lp(a), lipoprotein (a); Alt, glutamic-pyruvic transaminase; BUN, blood urea nitrogen; Cr, serum creatinine; UA, serum uric acid. Data are represented as mean (SD) or number (proportion), and the *p*-values are calculated using the Welch Two Sample *t*-test or Fisher’s exact test.

We used the createDataPartition function in the caret package to divide the training set and the test set, in which the test set accounted for 70% of all data. Continuous variables were normalized by subtracting the average value and dividing by the standard deviation.

In the realm of machine learning data preprocessing, the phenomenon of class imbalance arises frequently, wherein the count of samples within one category significantly surpasses that in the other. This incongruity can pose challenges for machine learning models, and its impact hinges on the relative proportions of samples in each category. To address this concern, this study applied a technique known as Synthetic Minority Over-sampling Technique for Nominal Continuous (SMOTE-NC) (Chawla et al., 2002) to the training set. This technique is specifically designed to handle the intricacies of imbalanced class data. SMOTE-NC serves as an extension of the Synthetic Minority Over-sampling Technique (SMOTE), distinguished by its competence in managing both nominal and continuous features. Within an imbalanced dataset, the SMOTE-NC algorithm orchestrates the generation of synthetic samples for the minority class through an oversampling mechanism involving interpolation of existing samples. A distinctive attribute of SMOTE-NC lies in its consideration of both continuous and nominal features, ensuring that the resultant synthetic samples faithfully capture the essence of the underlying data distribution.

### 2.3 Model building

We harnessed six prevalent classifiers to formulate a model aimed at discerning carotid artery plaque presence.

Logistic Regression (LR) is a machine learning algorithm specifically designed for binary classification tasks. It predicts the likelihood of a sample belonging to a particular category by mapping a linear combination of variables to a range between 0 and 1. LR allows for the interpretation of variable importance through the coefficients associated with variables in the model. Thanks to its relatively simple model structure, LR has low computational costs and can provide good predictive performance even with limited training data.

Support Vector Machines (SVM), a prominent machine learning approach for binary classification, adeptly discerns intricate boundaries within data. By strategically positioning a hyperplane, SVM achieves optimal separation between distinct classes while maximizing the margin between them. This strategy ensures robust generalization to new data by prioritizing pivotal samples—known as support vectors—that serve as anchor points for classification decisions. The potency of SVM resides in its capacity to accommodate complex relationships through the utilization of kernel functions. These functions facilitate data projection into higher-dimensional spaces, enabling linear separation even in scenarios where the original feature space may seem inseparable. Consequently, SVM stands as a formidable tool for distinguishing intricate patterns across various domains.

Artificial neural networks (ANNs), a pivotal machine learning architecture, exhibit a remarkable prowess in capturing intricate patterns and nuances within complex datasets. Operating akin to the intricate neural pathways of the human brain, ANNs encompass interconnected nodes, or neurons, each processing and transmitting information. In the realm of binary classification, ANNs navigate the realm of uncertainty by formulating a predictive framework that can discern whether a given sample pertains to a specific category. This intricate process entails multiple hidden layers and adaptable weights that iteratively adjust, culminating in a comprehensive comprehension of the underlying relationships within the data. The distinct strength of ANNs lies in their capacity to glean non-linear interactions and high-order dependencies among variables. Despite their computational demands and susceptibility to overfitting in certain scenarios, ANNs have proven their mettle in various domains, even when confronted with intricate classification tasks.

The Random Forest model stands as a robust machine learning tool, with its fundamental building block being the decision tree. Comprising multiple decision trees, each tree is trained on a randomized subset of data to mitigate the risk of overfitting. In the context of binary classification, the Random Forest model plays a pivotal role. It achieves this by aggregating the results of each decision tree’s predictions, thereby furnishing more resilient and accurate classification forecasts. Notably, due to its capacity to handle substantial features and samples while effectively capturing intricate relationships among features, the Random Forest model excels in intricate classification predicaments.

Extreme Gradient Boosting (XGBoost) stands as a potent and efficient machine learning algorithm extensively utilized across a spectrum of forecasting and classification tasks. Its foundational principle draws upon gradient boosting technology, involving iterative training of an ensemble of weak learners (typically decision trees), which are then amalgamated into a formidable ensemble model. Throughout each iteration, XGBoost seeks out novel models that yield maximal reduction in the loss function, all while judiciously considering the model’s complexity to stave off overfitting. In the context of binary classification, XGBoost shines with exceptional performance. It aptly handles high-dimensional, sparse feature spaces and achieves remarkable classification outcomes even amidst intricate inter-feature relationships. By optimizing split points and leaf node weights, XGBoost efficiently captures nonlinear associations inherent in data. Furthermore, XGBoost offers a feature importance evaluation, illuminating the features that contribute significantly to classification, thereby aiding feature engineering and model interpretation.

LightGBM, or the “Light Gradient Boosting Machine,” stands as an efficient and distributed machine learning model based on the gradient boosting algorithm. Similar to XGBoost, it employs iterative training of weak learners (typically decision trees), which are then amalgamated into a potent ensemble model. However, LightGBM has achieved substantial advancements in speed and memory utilization through innovative techniques and optimization strategies. In the realm of binary classification, LightGBM, akin to the XGBoost model, shines brightly. It employs histogram-based decision tree splitting methods, allowing for efficient handling of large-scale and high-dimensional data. Furthermore, LightGBM introduces the Leaf-wise growth strategy, which prioritizes splitting leaf nodes with larger gradients to accelerate model convergence. This renders LightGBM more efficient than XGBoost in certain scenarios, particularly when dealing with larger datasets.

In this study, before establishing each final machine learning model, it was imperative to construct a hyperparameter space encompassing a range of potential values for various hyperparameters. This approach aimed to encompass diverse combinations of model parameters, facilitating the quest for optimal performance. The aggregation of this hyperparameter space was subjected to a grid search strategy to iteratively explore parameter values. For each parameter combination, a ten-fold cross-validation was executed. Based on the cross-validation outcomes, the hyperparameter set exhibiting the best performance (with optimal AUC serving as the benchmark in this study) was chosen. All the optimal parameters and detailed information about the device and model construction environment can be found in Supplementary Table S2. Subsequently, the final machine learning model was solidified through comprehensive retraining on the entire training set.

### 2.4 Model performance assessment

We evaluate the predictive capabilities of various ML models by constructing confusion matrices and calculating metrics including Area Under the Curve (AUC), accuracy, sensitivity, specificity, F1 score, recall, precision, and kappa value. In this section, we provide an overview of the diverse metrics employed to assess the performance of machine learning models.

Accuracy serves as a pivotal assessment metric within the realm of machine learning, signifying the ratio of accurately predicted instances to the total number of instances. A heightened accuracy underscores enhanced classification efficacy.

$$\text{Accuracy}=\left(\text{TP}+\text{TN}\right)/\left(\text{TP}+\text{FP}+\text{TN}+\text{FN}\right)\times 100\%$$

where TP, TN, FP, and FN denote true positive, true negative, false positive, and false negative, respectively.

Sensitivity, commonly referred to as the true positive rate, stands as a pivotal performance metric within the purview of machine learning models. This metric quantifies the model’s competence in correctly pinpointing individuals who yield positive test results. Sensitivity gauges the fraction of actual positive instances that the model accurately recognizes as positive, thereby offering insights into the model’s aptitude for detecting true positives.

$$\text{Sensitivity}=\text{TP}/\left(\text{TP}+\text{FN}\right)\times 100\%$$

Specificity denotes the ratio of correctly identified negative cases to the total number of negative cases. This metric serves as an indicator of a machine learning model’s capability to accurately recognize instances that are negative. Increased specificity corresponds to a reduced false positive rate, thereby enhancing the precision of the model’s negative predictions. In essence, specificity measures the model’s effectiveness in distinguishing true negative cases.

$$\text{Specificity}=\text{TN}/\left(\text{TN}+\text{FP}\right)\times 100\%$$

Positive Predictive Value (PPV) represents the proportion of correctly predicted positive samples out of all samples predicted as positive by the model. A higher PPV indicates that the model is more accurate when predicting the positive class, with relatively fewer occurrences of misclassifying negative class samples as positive. This reflects the model’s classification effectiveness.

$$\text{PPV}=\text{TP}/\left(\text{TP}+\text{FP}\right)\times 100\%$$

Negative Predictive Value (NPV) represents the proportion of samples correctly predicted as the negative class out of all samples predicted as the negative class by the model. NPV is typically used to assess the model’s performance in excluding the negative class.

$$\text{NPV}=\text{TN}/\left(\text{TN}+\text{FN}\right)\times 100\%$$

F1 score is a balanced measure that amalgamates both PPV and sensitivity. In the context of the classification task at hand, it delineates the model’s capability to concurrently consider both true positive predictions and false positives, thus providing a holistic insight into its performance.

$$F1\text{ score}=2*\left(\text{PPV }*\text{Sensitivity}\right)/\left(\text{PPV}+\text{Sensitivity}\right)$$

Kappa stands as a statistical metric for agreement, encompassing values within the range of −1 to 1. In the context of the classification quandary being examined, kappa quantifies the level of concordance between the outcomes predicted by the model and the factual classification outcomes. Generally, a higher kappa value is construed as emblematic of heightened concurrence between the classifier’s predictions and the authentic outcomes.

$$\text{Kappa}=\left(\text{Po}-\text{Pe}\right)/\left(1-\text{Pe}\right)$$

where Po signifies the observed agreement proportion between the two classifiers, and Pe denotes the anticipated agreement proportion stemming from chance.

Receiver Operating Characteristic (ROC) curves provide a visual depiction of a binary classifier system’s performance. These curves are formed by plotting the true positive rate (TPR) against the false positive rate (FPR) across different threshold settings. An ideal classifier boasts an ROC curve tracing through the upper left corner of the graph, signifying high TPR and low FPR. The Area Under the Curve (AUC) quantifies the classifier’s capacity to differentiate between positive and negative classes.

Root Mean Square Error (RMSE) is a metric used to measure the error between predicted values of a regression model and actual observed values. It represents the square root of the average squared difference between predicted and actual values. A smaller RMSE indicates a smaller prediction error and better fitting of the model. In this study, RMSE after Permutations is utilized to rank the feature importance of all models. The underlying concept involves randomly shuffling the order of feature values and then comparing the root mean square errors of the model on both the original and permuted data. This method aids in assessing the contribution of each feature to the model’s performance, facilitating the ranking of features to determine their relative importance.

## 3 Model performance assessment

### 3.1 Characteristics and distribution of participants

**FIGURE 2:**
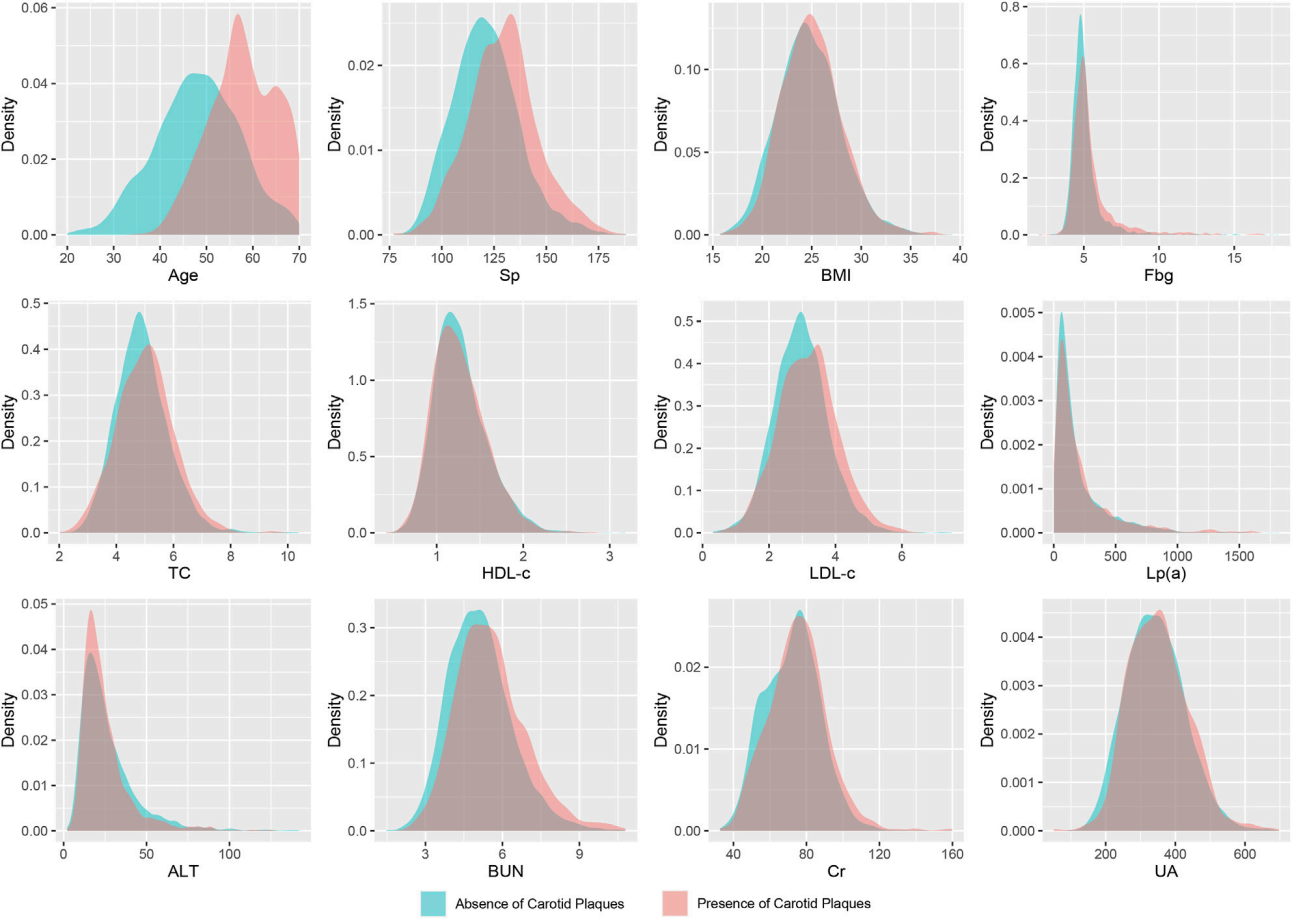
Density distribution curves of all variables. Sp, systolic pressure; BMI, body mass index; Fbg, fasting blood-glucose; TC, total cholesterol; HDL-c, high-density lipoprotein cholesterol; LDL-c, low-density lipoprotein cholesterol; Lp(a), lipoprotein (a); Alt, glutamic-pyruvic transaminase; BUN, blood urea nitrogen; Cr, serum creatinine; UA, serum uric acid.

A total of 5,211 participants were ultimately included in this study, comprising 3,724 males and 1,487 females. Among the participants, 1,164 individuals were diagnosed with carotid artery plaques. Their average age was 57.88 ± 7.07 years, while those without carotid artery plaques had an average age of 48.11 ± 9.11 years. All included variables were presented as mean (SD), and a two-sample *t*-test revealed significant differences between the two groups (Figure 2; Table 1).

### 3.2 Model performance

**FIGURE 3:**
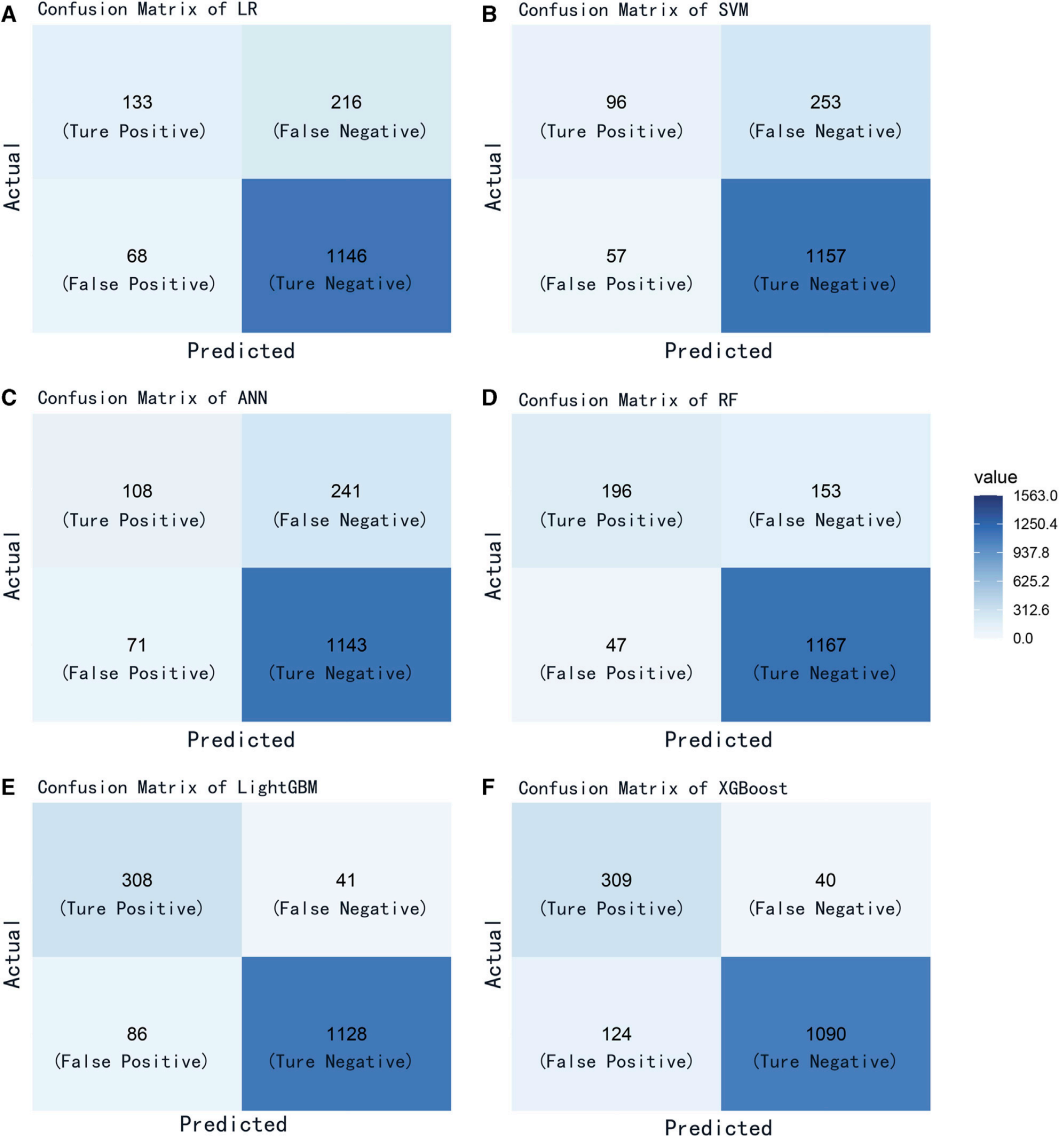
Confusion matrix of all models. **(A)** logistic regression; **(B)** support vector machine; **(C)** artificial neural network; **(D)** random forest; **(E)** light gradient boosting machine; **(F)** extreme gradient boosting.

**FIGURE 4:**
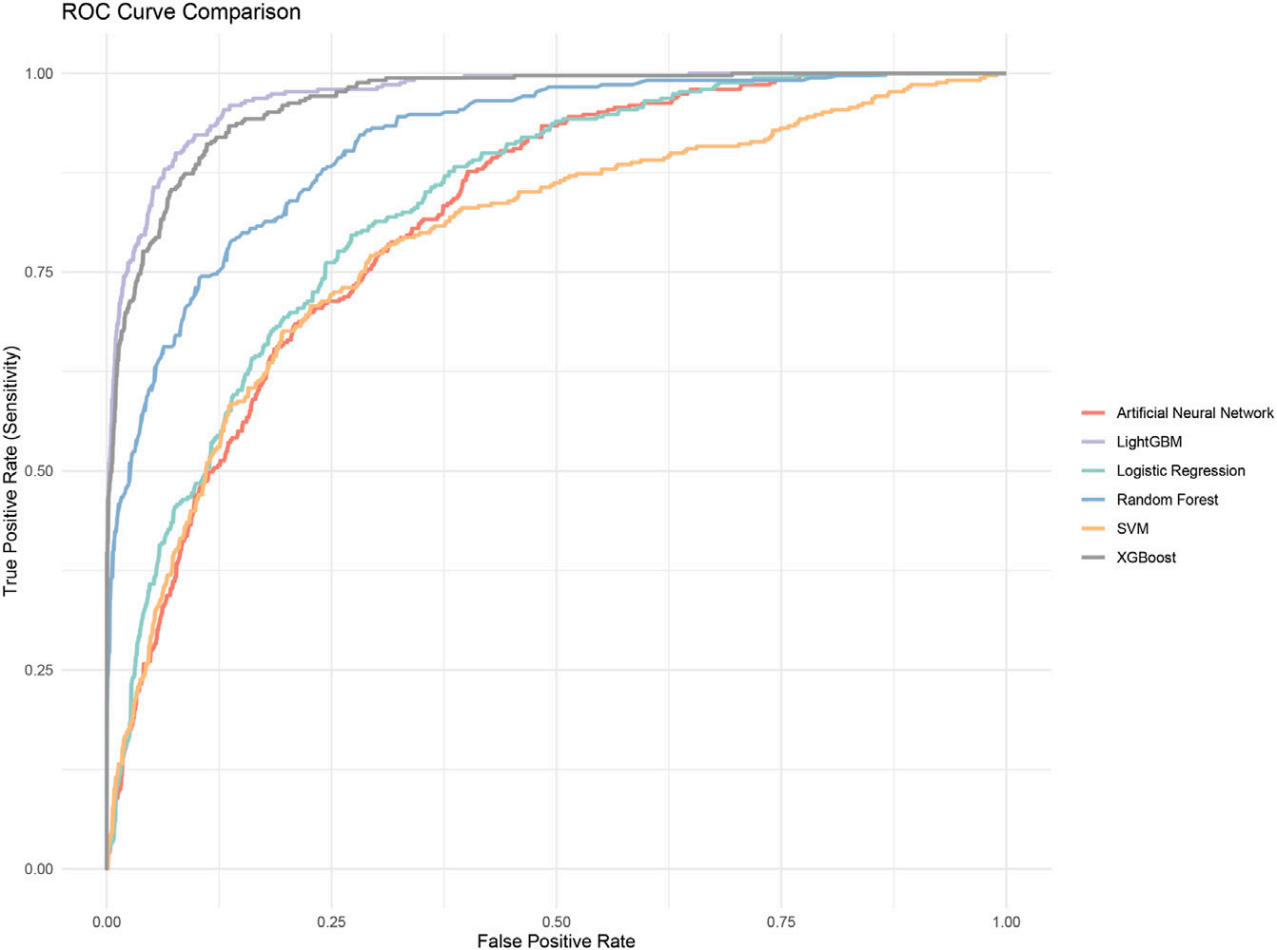
ROC curves of all models.

**FIGURE 5:**
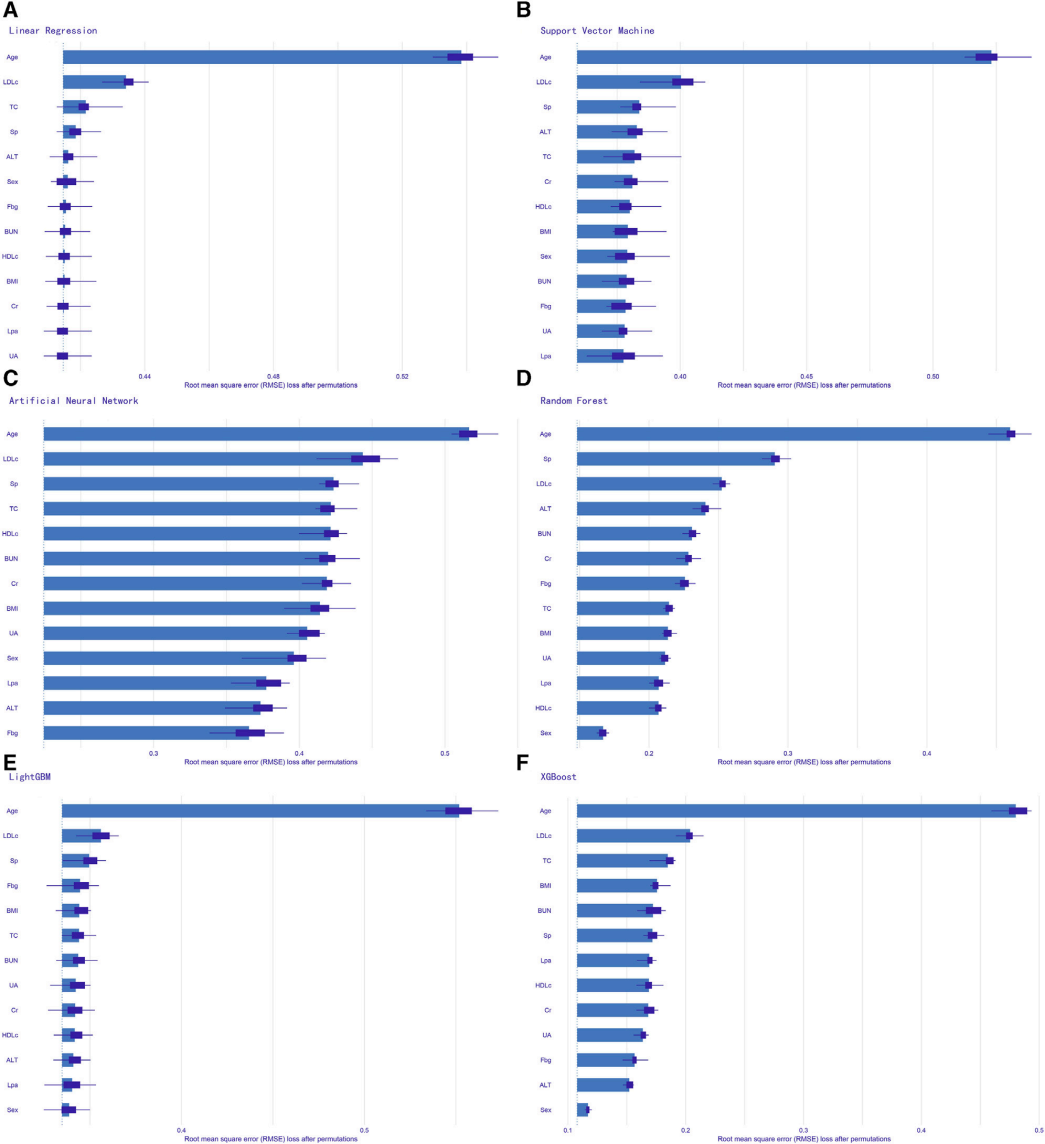
Feature importance analysis of all models. **(A)** logistic regression; **(B)** support vector machine; **(C)** artificial neural network; **(D)** random forest; **(E)** light gradient boosting machine; **(F)** extreme gradient boosting. Sp, systolic pressure; BMI, body mass index; Fbg, fasting blood-glucose; TC, total cholesterol; HDLc, high-density lipoprotein cholesterol; LDLc, low-density lipoprotein cholesterol; Lp(a), lipoprotein (a); Alt, glutamic-pyruvic transaminase; BUN, blood urea nitrogen; Cr, serum creatinine; UA, serum uric acid.

We evaluated the performance of the machine learning models by constructing confusion matrices and ROC curves. The consolidated confusion matrices and ROC curves for all models are illustrated in Figures 3, 4, respectively. Through comparison of the six constructed ML models, it was observed that LightGBM exhibited the highest accuracy in predicting carotid artery plaques at 91.8% and boasted the highest AUC (97.23%). XGBoost closely followed LightGBM in terms of prediction accuracy, with an AUC of 96.52%, signifying XGBoost’s + as one of the top predictive models. Both XGBoost and LightGBM attained kappa values exceeding 70%, suggesting strong repeatability of the models. While logistic regression, SVM, and artificial neural network models achieved accuracy rates above 80%, their sensitivity and kappa values were comparatively lower. This implies that despite employing SMOTE-NC to balance the data, these three algorithms exhibited limitations in identifying positive samples, leading to reduced repeatability of their predictions (Table 2). Based on the results of feature importance analysis, it was evident that age, LDL-c, and systolic blood pressure played significant roles across the majority of the models (Figure 5).

**TABLE 2:** Assessment of six machine learning models.

Model	Accuracy (%)	Sensitivity (%)	Specificity (%)	PPV (%)	NPV (%)	F1 score (%)	Kappa (%)	AUC (%)
LR	81.8298	38.1089	94.3987	66.1692	84.1410	48.3636	38.2928	83.4146
SVM	80.1663	27.5072	95.3048	62.7451	82.0567	38.2470	28.5177	78.8999
ANN	80.0384	30.9456	94.1516	60.3352	82.5867	40.9091	30.3669	81.7426
RF	87.2041	56.1605	96.1285	80.6584	88.4091	66.2162	58.6334	91.3072
LightGBM	91.8746	88.2521	92.9160	78.1726	96.4927	82.9071	77.6033	97.2329
XGBoost	89.5074	88.5387	89.7858	71.3626	96.4602	79.0281	72.1388	96.5238

LR, logistic regression; SVM, support vector machine; ANN, artificial neural network; RF, random forest; LightGBM, light gradient boosting machine; XGBoost, extreme gradient boosting; PPV, positive predictive value; NPV, negative predictive value; AUC, area under curve of test set.

## 4 Discussion

This study encompasses clinical data from 5,211 participants and employs six ML models, thus establishing itself as the most extensive machine learning investigation to predict carotid artery plaques in the Chinese population based on health examinations and blood biochemical indicators. The findings underscore the efficacy of ML in effectively predicting the occurrence of carotid artery plaques, with the LightGBM model emerging as the most proficient predictor among all machine learning models. Our assessment of the various machine learning models in the results section reveals that not only the LightGBM model but also the XGBoost model and the random forest model exhibit high accuracy. Moreover, all three models boast AUC values exceeding 90%, indicating their ability to effectively discriminate between positive and negative cases across varying thresholds. This reflects substantial overlap in the probability distributions of the models’ predictions for positive and negative cases, showcasing robust classification capability. Furthermore, the elevated Kappa values for these models denote excellent repeatability. This is likely attributed to their shared ensemble learning framework, wherein these models amalgamate the predictive outcomes of multiple weak learners (decision trees) to construct more potent models, ultimately enhancing overall performance. It’s worth noting that both LightGBM and XGBoost incorporate regularization mechanisms to prevent overfitting, further enhancing their generalization capability. These advantages position ensemble learning models, particularly LightGBM and XGBoost, as highly accurate tools for binary classification prediction and exemplary performers in diagnosing a wide range of clinical conditions.

Compared to previous machine learning study (Wu et al., 2022) that used physical examinations and biochemical indicators to predict carotid artery plaques in the Chinese population, our study has several advantages. Firstly, our study boasts a much larger sample size, approximately three times larger than previous research. Additionally, the ratio of cases to controls in our study aligns more closely with the predicted prevalence of carotid artery plaques in the Chinese population based on prior research (Song et al., 2018). While this might introduce some data imbalance issues affecting model construction, we addressed this problem to some extent by employing SMOTE-NC for data balancing. Secondly, considering the trend of a younger onset of atherosclerosis (Libby, 2021), and recognizing carotid artery plaques as a sentinel manifestation of atherosclerosis, we expanded the age range for screening beyond the traditional 40 and above demographic. Our screening population is now targeted at individuals aged 18 to 70, providing a broader coverage. Lastly, concerning the predictive outcomes of our models, we obtained results that are similar to previous research using the XGBoost model. Surprisingly, we found that LightGBM, compared to the XGBoost model, demonstrated higher accuracy and superior robustness. It appears that LightGBM may excel in this predictive task.

The Tromsø Study conducted back in the 1970s demonstrated that the prevalence of carotid plaques increases with age, regardless of gender (Joakimsen et al., 1999). In recent years, a small-scale cohort study (Lu et al., 2019) utilizing high-resolution magnetic resonance imaging for diagnosing carotid plaques confirmed that compared to patients under 60 years old, those aged 60–75 and even above 75 exhibited a greater annual change in carotid artery wall volume and maximum wall area. Furthermore, it was established that age is independently associated with the progression of carotid plaques. Our baseline data density distribution and feature table also reveal discernible differences in the density distribution of individuals with and without plaques in the age variable. This aligns with the observed highest feature importance of age across all models in our study. The irreversible physiological process of aging significantly impacts metabolic and physiological changes. Signaling pathways linked to aging influence endothelial function, smooth muscle function within blood vessels, and the structural integrity of arterial walls. These processes render arteries more susceptible to atherosclerosis. This foundational biomedical perspective offers an explanation for the closely linked relationship between advancing age and an elevated incidence of carotid plaques. A cohort study (Lu et al., 2019) conducted on a Chinese population suggests that the 5-year change in systolic blood pressure is associated with the occurrence of carotid atherosclerotic plaques. This correlation persists even after adjusting for gender, age, smoking status, blood lipid levels, and intima-media thickness of the carotid artery. Foundational research (Selvin et al., 2005) indicates that the altered shear stress caused by hypertension and increased oxidative stress can disrupt the structural integrity of the endothelium, leading to increased vascular permeability. This, in turn, promotes the infiltration of adhesive molecules, recruitment of chemotactic agents to the intima, and an increase in pro-inflammatory cytokines, ultimately contributing to the development of atherosclerotic lesions. The conclusions from clinical and foundational studies also indirectly support the rationale behind the higher feature importance of systolic blood pressure observed in this study. The extensive body of evidence from hundreds of prospective cohort studies, Mendelian randomization studies, and randomized trials consistently indicates a dose-dependent, logarithmic relationship between the absolute magnitude of LDLc (Low-Density Lipoprotein cholesterol) exposure and ASCVD (Atherosclerotic Cardiovascular Disease) risk. Importantly, this association is not merely correlational but is considered to be causal (Ference et al., 2017). The prevailing mechanistic view suggests that LDL contributes to the development of atherosclerosis primarily through the retention of LDL beneath the arterial intima, where it undergoes oxidative modifications, leading to the formation of oxidized LDL (oxLDL). Macrophages then engulf oxLDL to form foam cells, which eventually merge to create atherosclerotic plaques (Borén et al., 2020). Carotid artery plaques, as a significant manifestation of atherosclerosis, are also strongly associated with elevated LDLc levels (Kim, 2021). Based on the clinical research evidence and the underlying physiological mechanisms mentioned above, it’s evident that in this study, age, systolic blood pressure, and LDLc levels exhibit higher feature importance and are well-supported by comprehensive evidence from the field of evidence-based medicine.

This study still has several limitations. Firstly, while it included over 5,000 samples, these samples were all from the Xiangya Second Hospital of Central South University. The representativeness of the population may not be as comprehensive as multicenter clinical studies. Additionally, the model’s generalization ability across different ethnic groups is questionable because it was validated by using 30% of the data from all sources as the test set instead of external data. Secondly, the ultrasound results in this study were performed by two qualified imaging centers’ attending physicians, and different results were determined by senior physicians. The collected carotid plaque diagnostic results in this study were the final results, not the individual diagnoses of different doctors. The consistency of diagnosis by doctors cannot be evaluated through the calculation of the kappa value. Since ultrasound diagnosis involves subjectivity, it may have some influence on the final diagnosis. Lastly, we only included baseline physical examination data and blood biochemical indicators. Some personal and past history that are often related to carotid plaques (Tsao et al., 2023), such as smoking status (Dempsey et al., 1990), atrial fibrillation (Chen et al., 2016), and diabetes (Yahagi et al., 2017; Gan et al., 2019), were not included in the analysis.

## 5 Conclusion

In our investigation, we unveiled that ensemble learning machine learning models, notably LightGBM, exhibit the capability to predict the presence of carotid artery plaques using variables such as gender, age, physical assessments, and blood biochemical markers. This approach showcases remarkable precision and robust repeatability. Among the 13 predictive factors, age, systolic blood pressure, and LDLc levels emerged as particularly influential across most models. Leveraging these machine learning models, healthcare practitioners can assess carotid artery plaque status even in the absence of carotid ultrasound, relying on routine physical examinations and blood biochemical markers. This breakthrough opens up new avenues for primary ischemic stroke prevention. In forthcoming research, our aim is to refine the model’s performance by gathering data from diverse populations to enhance its applicability.

## Data Availability

The original contributions presented in the study are included in the article/Supplementary Material, further inquiries can be directed to the corresponding authors.

## References

[B1] Borén J. Chapman M. J. Krauss R. M. Packard C. J. Bentzon J. F. Binder C. J. (2020). Low-density lipoproteins cause atherosclerotic cardiovascular disease: pathophysiological, genetic, and therapeutic insights: a consensus statement from the European Atherosclerosis Society Consensus Panel. Eur. Heart J. 41 (24), 2313–2330. eng. Epub 2020/02/14. Cited in: Pubmed; PMID 32052833. 10.1093/eurheartj/ehz962 32052833 PMC7308544

[B2] Chawla N. V. Bowyer K. W. Hall L. O. Kegelmeyer W. P. (2002). SMOTE: synthetic minority over-sampling technique. J. Artif. Intell. Res. 16, 321–357. 10.1613/jair.953

[B3] Chen L. Y. Leening M. J. Norby F. L. Roetker N. S. Hofman A. Franco O. H. (2016). Carotid intima-media thickness and arterial stiffness and the risk of atrial fibrillation: the atherosclerosis risk in communities (ARIC) study, multi-ethnic study of atherosclerosis (MESA), and the rotterdam study. J. Am. Heart Assoc. 5 (5). eng. Epub 2016/05/22. Cited in: Pubmed; PMID 27207996. 10.1161/jaha.115.002907 PMC4889172 27207996

[B4] Chinese Society of Health Management, Chinese Society of Ultrasound in Medicine CSoC (2015). Guidelines for carotid ultrasound examination in the Chinese health examination population. Chin. J. Health Manag. 9 (4), 7. 10.3760/cma.j.issn.1674-0815.2015.04.004

[B5] Dempsey R. J. Diana A. L. Moore R. W. (1990). Thickness of carotid artery atherosclerotic plaque and ischemic risk. Neurosurgery 27 (3), 343–348. eng. Epub 1990/09/01. Cited in: Pubmed; PMID 2234325. 10.1097/00006123-199009000-00001 2234325

[B6] Fan J. Li X. Yu X. Liu Z. Jiang Y. Fang Y. (2023). Global burden, risk factor analysis, and prediction study of ischemic stroke, 1990-2030. Neurology 101 (2), e137–e150. eng. Epub 2023/05/18. Cited in: Pubmed; PMID 37197995. 10.1212/wnl.0000000000207387 37197995 PMC10351546

[B7] Ference B. A. Ginsberg H. N. Graham I. Ray K. K. Packard C. J. Bruckert E. (2017). Low-density lipoproteins cause atherosclerotic cardiovascular disease. 1. Evidence from genetic, epidemiologic, and clinical studies. A consensus statement from the European Atherosclerosis Society Consensus Panel. Eur. Heart J. 38 (32), 2459–2472. eng. Epub 2017/04/27. Cited in: Pubmed; PMID 28444290. 10.1093/eurheartj/ehx144 28444290 PMC5837225

[B8] Gan W. Bragg F. Walters R. G. Millwood I. Y. Lin K. Chen Y. (2019). Genetic predisposition to type 2 diabetes and risk of subclinical atherosclerosis and cardiovascular diseases among 160,000 Chinese adults. Diabetes 68 (11), 2155–2164. eng. Epub 2019/08/11. Cited in: Pubmed; PMID 31399431. 10.2337/db19-0224 31399431 PMC6804628

[B9] GBD 2019 Stroke Collaborators (2021). Global, regional, and national burden of stroke and its risk factors, 1990-2019: a systematic analysis for the Global Burden of Disease Study 2019. Lancet Neurol. 20 (10), 795–820. eng. Epub 2021/09/07. Cited in: Pubmed; PMID 34487721. 10.1016/s1474-4422(21)00252-0 34487721 PMC8443449

[B10] Joakimsen O. Bonaa K. H. Stensland-Bugge E. Jacobsen B. K. (1999). Age and sex differences in the distribution and ultrasound morphology of carotid atherosclerosis: the Tromsø Study. Arterioscler. Thromb. Vasc. Biol. 19 (12), 3007–3013. eng. Epub 1999/12/11. Cited in: Pubmed; PMID 10591682. 10.1161/01.atv.19.12.3007 10591682

[B11] Joshi R. D. Dhakal C. K. (2021). Predicting type 2 diabetes using logistic regression and machine learning approaches. Int. J. Environ. Res. Public Health 18 (14). eng. Epub 2021/07/25. Cited in: Pubmed; PMID 34299797. 10.3390/ijerph18147346 PMC8306487 34299797

[B12] Kim J. S. (2021). Role of blood lipid levels and lipid-lowering therapy in stroke patients with different levels of cerebral artery diseases: reconsidering recent stroke Guidelines. J. Stroke 23 (2), 149–161. eng. Epub 2021/06/10. Cited in: Pubmed; PMID 34102752. 10.5853/jos.2021.01249 34102752 PMC8189863

[B13] Latha S. Muthu P. Lai K. W. Khalil A. Dhanalakshmi S. (2021). Performance analysis of machine learning and deep learning architectures on early stroke detection using carotid artery ultrasound images. Front. Aging Neurosci. 13, 828214. eng. Epub 2022/02/15. Cited in: Pubmed; PMID 35153728. 10.3389/fnagi.2021.828214 35153728 PMC8830903

[B14] Lekadir K. Galimzianova A. Betriu A. Del Mar Vila M. Igual L. Rubin D. L. (2017). A convolutional neural network for automatic characterization of plaque composition in carotid ultrasound. IEEE J. Biomed. Health Inf. 21 (1), 48–55. eng. Epub 2016/11/29. Cited in: Pubmed; PMID 27893402. 10.1109/jbhi.2016.2631401 PMC5293622 27893402

[B15] Libby P. (2021). The changing landscape of atherosclerosis. Nature 592 (7855), 524–533. eng. Epub 2021/04/23. Cited in: Pubmed; PMID 33883728. 10.1038/s41586-021-03392-8 33883728

[B16] Lorenz M. W. Markus H. S. Bots M. L. Rosvall M. Sitzer M. (2007). Prediction of clinical cardiovascular events with carotid intima-media thickness: a systematic review and meta-analysis. Circulation 115 (4), 459–467. eng. Epub 2007/01/24. Cited in: Pubmed; PMID 17242284. 10.1161/circulationaha.106.628875 17242284

[B17] Lu M. Peng P. Qiao H. Cui Y. Ma L. Cui B. (2019). Association between age and progression of carotid artery atherosclerosis: a serial high resolution magnetic resonance imaging study. Int. J. Cardiovasc Imaging 35 (7), 1287–1295. eng. Epub 2019/02/11. Cited in: Pubmed; PMID 30739271. 10.1007/s10554-019-01538-4 30739271

[B18] Prati P. Tosetto A. Vanuzzo D. Bader G. Casaroli M. Canciani L. (2008). Carotid intima media thickness and plaques can predict the occurrence of ischemic cerebrovascular events. Stroke 39 (9), 2470–2476. eng. Epub 2008/07/12. Cited in: Pubmed; PMID 18617662. 10.1161/strokeaha.107.511584 18617662

[B19] Qi W. Ma J. Guan T. Zhao D. Abu-Hanna A. Schut M. (2020). Risk factors for incident stroke and its subtypes in China: a prospective study. J. Am. Heart Assoc. 9 (21), e016352. eng. Epub 2020/10/27. Cited in: Pubmed; PMID 33103569. 10.1161/jaha.120.016352 33103569 PMC7763402

[B20] Saba L. Nardi V. Cau R. Gupta A. Kamel H. Suri J. S. (2022). Carotid artery plaque calcifications: lessons from histopathology to diagnostic imaging. Stroke 53 (1), 290–297. eng. Epub 2021/11/11. Cited in: Pubmed; PMID 34753301. 10.1161/strokeaha.121.035692 34753301

[B21] Selvin E. Coresh J. Shahar E. Zhang L. Steffes M. Sharrett A. R. (2005). Glycaemia (haemoglobin A1c) and incident ischaemic stroke: the atherosclerosis risk in communities (ARIC) study. Lancet Neurol. 4 (12), 821–826. eng. Epub 2005/11/22. Cited in: Pubmed; PMID 16297840. 10.1016/s1474-4422(05)70227-1 16297840

[B22] Song P. Xia W. Zhu Y. Wang M. Chang X. Jin S. (2018). Prevalence of carotid atherosclerosis and carotid plaque in Chinese adults: a systematic review and meta-regression analysis. Atherosclerosis 276, 67–73. eng. Epub 2018/07/24. Cited in: Pubmed; PMID 30036743. 10.1016/j.atherosclerosis.2018.07.020 30036743

[B23] Tsao C. W. Aday A. W. Almarzooq Z. I. Anderson C. A. M. Arora P. Avery C. L. (2023). Heart disease and stroke statistics-2023 update: a report from the American heart association. Circulation 147 (8), e93–e621. eng. Epub 2023/01/26. Cited in: Pubmed; PMID 36695182. 10.1161/cir.0000000000001123 36695182 PMC12135016

[B24] Weng S. Hu D. Chen J. Yang Y. Peng D. (2023). Prediction of fatty liver disease in a Chinese population using machine-learning algorithms. Diagn. (Basel) 13 (6). eng. Epub 2023/03/30. Cited in: Pubmed; PMID 36980476. 10.3390/diagnostics13061168 PMC10047083 36980476

[B25] Wu D. Cui G. Huang X. Chen Y. Liu G. Ren L. (2022). An accurate and explainable ensemble learning method for carotid plaque prediction in an asymptomatic population. Comput. Methods Programs Biomed. 221, 106842. eng. Epub 2022/05/16. Cited in: Pubmed; PMID 35569238. 10.1016/j.cmpb.2022.106842 35569238

[B26] Yahagi K. Kolodgie F. D. Lutter C. Mori H. Romero M. E. Finn A. V. (2017). Pathology of human coronary and carotid artery atherosclerosis and vascular calcification in diabetes mellitus. Arterioscler. Thromb. Vasc. Biol. 37 (2), 191–204. eng. Epub 2016/12/03. Cited in: Pubmed; PMID 27908890. 10.1161/atvbaha.116.306256 27908890 PMC5269516

