# Supplementary material for: Utilizing machine learning algorithms for the prediction of carotid artery plaques in a Chinese population
Source: Front Physiol. 2023 Nov 6;14:1295371. doi: 10.3389/fphys.2023.1295371 (PMC10657816; doi:10.3389/fphys.2023.1295371)
Supplement: Supplementary file 2 [file DataSheet1.docx]

Supplementary Material

# Supplementary Figures


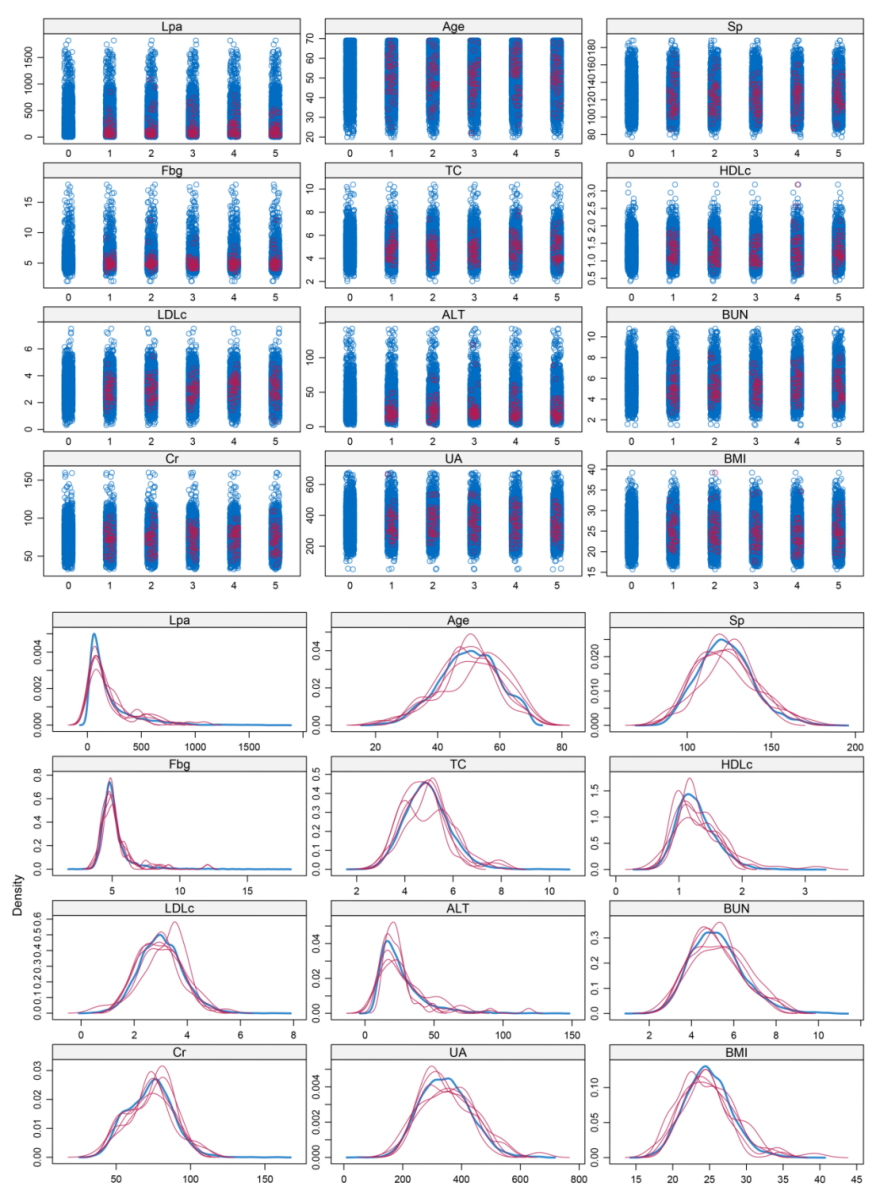


**Supplementary Figure 1.** The blue circles or blue curves in the graph represent the original data and the density distribution curve of the original data, respectively. The red circles or red curves represent the imputed data and the density distribution curve of the imputed data, respectively.

Sp: systolic pressure; BMI: body mass index; Fbg: fasting blood-glucose; TC: total cholesterol; HDL-c: high-density lipoprotein cholesterol; LDL-c: low-density lipoprotein cholesterol; Lp(a): lipoprotein (a); Alt: glutamic-pyruvic transaminase; BUN: blood urea nitrogen; Cr: serum creatinine; UA : serum uric acid.

# Supplementary Tables

| **Machine Learning Models** | **Optimal Hyperparameters and Model Details** |
| --- | --- |
| Logistic Regression | Hyperparameters: family : “binomial”  link: “logit”  alpha: 0.3  lamda:0.4 |
| Support Vector Machines | Hyperparameters: kernel: “radial”  cost:1  gamma:0.75 |
| Artificial neural networks | Model Type: Feedforward Neural Network |
|  | Model Structure: Input Layer: 13 neurons  Hidden Layer 1: 32 neurons (ReLU)  Hidden Layer 2: 16 neurons (ReLU)  Output Layer: 1 neuron (Sigmoid) |
|  | Hyperparameters: optimizer: “adam”  loss function: “binary_crossentropy”  metrics: roc_auc  batch size: 60  epochs: 150 |
| Random Forest | Hyperparameters: ntree: 650  mtry: 4  nodesize: 5 |
| LightGBM | Hyperparameters: objective: “binary”  boosting_type: “gbdt”  num_leaves: 25  learning_rate: 0.15  max_depth: 6  num_iterations: 125 |
| Extreme Gradient Boosting | Hyperparameters: objective: “binary:logistic”  eta:0.1  max_depth: 6  gamma: 0.2  colsample_bytree: 1  min_child_weight: 1  subsample: 1 |

**Supplementary Table 1.** This table describes various machine learning models that underwent a grid search strategy, iteratively exploring parameter values. For each parameter combination, 10-fold cross-validation was performed. Based on the cross-validation results, the table displays the best-performing hyperparameter sets (with the best AUC as the benchmark in this study).

Data preprocessing and model construction were performed using R language (version 4.1.3), Python (version 3.9.7), and TensorFlow (version 2.5.0). The model training process took place on a computer equipped with an NVIDIA RTX 3070 Ti GPU, 64 GB of RAM, and an 11th Gen Intel(R) Core(TM) i5-11400 @ 2.60GHz CPU. The operating system used was Windows 10 Professional 64-bit (Version 21H1 / DirectX 12).
